# MYC overexpression and SMARCA4 loss cooperate to drive medulloblastoma formation in mice

**DOI:** 10.1186/s40478-023-01654-2

**Published:** 2023-11-02

**Authors:** Carolin Göbel, Shweta Godbole, Melanie Schoof, Dörthe Holdhof, Catena Kresbach, Carolin Loose, Julia Neumann, Ulrich Schüller

**Affiliations:** 1 Department of Pediatric Hematology and Oncology, University Medical Center Hamburg-Eppendorf, Martinistrasse 52, Hamburg, 20251 Germany; 2 Research Institute Children’s Cancer Center Hamburg, Martinistrasse 52, Building N63 (LIV), Hamburg, D-20251 Germany; 3 Center for Molecular Neurobiology, Falkenried 94, Hamburg, 20251 Germany; 4 Institute of Neuropathology, University Medical Center Hamburg-Eppendorf, Martinistrasse 52, Hamburg, 20251 Germany

**Keywords:** Group 3 medulloblastoma, MYC, BRG1, BAF complex, Chromatin remodeling

## Abstract

Group 3 medulloblastoma is one of the most aggressive types of childhood brain tumors. Roughly 30% of cases carry genetic alterations in *MYC*, *SMARCA4*, or both genes combined. While overexpression of MYC has previously been shown to drive medulloblastoma formation in mice, the functional significance of *SMARCA4* mutations and their suitability as a therapeutic target remain largely unclear. To address this issue, we combined overexpression of MYC with a loss of SMARCA4 in granule cell precursors. Both alterations did not increase proliferation of granule cell precursors in vitro. However, combined MYC overexpression and SMARCA4 loss successfully induced tumor formation in vivo after orthotopic transplantation in recipient mice. Resulting tumors displayed anaplastic histology and exclusively consisted of SMARCA4-negative cells although a mixture of recombined and non-recombined cells was injected. These observations provide first evidence for a tumor-promoting role of a SMARCA4 deficiency in the development of medulloblastoma. In comparing the transcriptome of tumors to the cells of origin and an established Sonic Hedgehog medulloblastoma model, we gathered first hints on deregulated gene expression that could be specifically involved in SMARCA4/MYC driven tumorigenesis. Finally, an integration of RNA sequencing and DNA methylation data of murine tumors with human samples revealed a high resemblance to human Group 3 medulloblastoma on the molecular level. Altogether, the development of SMARCA4-deficient medulloblastomas in mice paves the way to deciphering the role of frequently occurring *SMARCA4* alterations in Group 3 medulloblastoma with the perspective to explore targeted therapeutic options.

**Supplementary Information:**

The online version contains supplementary material available at 10.1186/s40478-023-01654-2.

## Introduction

The BAF (BRG1/BRM-associated factor) chromatin remodeling complex greatly influences gene expression in mammals by regulating accessibility of DNA regions for the binding of transcription factors [2, 12]. Its catalytic activity depends on the presence of one of the mutually exclusive ATPase subunits SMARCA2 or SMARCA4 (SWI/SNF related, matrix associated, actin dependent regulator of chromatin, subfamily A, member 4; also known as BRG1 [BRAHMA related gene 1]) [31, 39]. In contrast to SMARCA2, SMARCA4 has proven indispensable in embryonic development as *Smarca4*-deficient mouse blastocysts die during the peri-implantation stage [8, 53]. Moreover, several mouse models have confirmed that functional SMARCA4 is essential for cerebellar development, neurogenesis, and gliogenesis [24, 25, 40, 43]. Consequently, alterations in the *SMARCA4* gene have been associated with a variety of intellectual disorders such as the Coffin-Siris syndrome and autism spectrum disorders [6, 68]. Moreover, deleterious *SMARCA4* mutations can be found throughout various cancer entities, including small cell carcinoma of the ovary, hypercalcemic type (SCCOHT), non-small cell lung cancer (NSCLC), pancreatic cancer, hepatocellular carcinoma, head and neck cancer, and atypical/teratoid rhabdoid tumors (ATRT) [15, 23, 27, 52]. In NSCLC and ATRT, *SMARCA4* alterations are associated with a significantly worse prognosis than *SMARCA4* wild-type cases [15, 20]. Medulloblastomas (MB), the most common malignant pediatric brain tumors, mainly show somatic heterozygous missense mutations of *SMARCA4*, which are suggested to have a dominant-negative effect resulting in a loss of function [15, 29, 44, 55]. MB can be divided into four main molecular subgroups according to their transcriptome and global DNA methylation: Sonic Hedgehog (SHH), Wingless/Int-1 (WNT), Group 3, and Group 4 [9, 65]. Alterations of *SMARCA4* mostly affect WNT and Group 3 MB, occurring in around 20% and 9–15% of cases, respectively, which places it among the most frequently mutated genes in both subgroups [17, 44]. However, the functional significance of these *SMARCA4* alterations in tumor development remains unknown. In this study, we focused on Group 3 MB, which mostly affect younger children and infants and show the worst prognosis of all subgroups with a median 5-year survival below 60% [10, 17, 30, 65]. Therefore, effective treatment regimens including targeted therapies are urgently needed. Besides *SMARCA4* mutations, recurrent alterations in Group 3 MB include *MYC* amplifications in 15–20% of cases, which correlate with poor survival [10, 17, 34]. *MYC* and *SMARCA* can also be concurrently altered as detected in around 1–6% of Group 3 MB [17, 29, 44, 55]. Several mouse models have convincingly demonstrated a tumor-driving role of MYC in the development of Group 3 MB [5, 32, 33, 47, 64]. However, none of the previously developed Group 3 MB mouse models include alterations of *Smarca4*. In this study, we present a new MB mouse model with combined MYC overexpression and SMARCA4 loss in granule cell precursors (GCPs) and provide evidence for a tumor-promoting role of a SMARCA4 deficiency in MB.

## Materials and methods

### Transgenic animals

All experimental procedures on animals were approved by the Government of Hamburg, Germany (N113/16, N050/2018, N099/2019) and were performed according to national regulations. Mice were kept on a 12 h dark/light cycle, and water and food were available *ad libitum*. Animals of both sexes were used for experiments. The strain *Smarca4*^*fl/fl*^ (also known as *Brg1*^*fl/fl*^) has been previously generated and described [28, 62], *Math1-creER*^*T2*^ mice were obtained from Jackson Laboratories, ME, USA (#7684) [37], and *CD1*^*nu/nu*^ mice were obtained from Charles River Laboratories, MA, USA (#086) [45]. *Math1creER*^*T2*^ and *Smarca4*^*fl/fl*^ mice were maintained on a *C57Bl6/J* background. Genotyping was performed by PCR using genomic DNA from ear or tail biopsies with the following primer pairs (5’-3’): *cre* (fw): TCCGGGCTGCCACGACCAA, *cre* (rv): GGCGCGGCAACACCATTTT, *Smarca4* floxed (fw): GTCATACTTATGTCATAGCC, *Smarca4* floxed (rv): GCCTTGTCTCAAACTGATAAG, *Smarca4* recombined (fw): GATCAGCTCATGCCCTAAGG, *Smarca4* recombined (rv): GCCTTGTCTCAAACTGATAAG. To induce *Smarca4* recombination in *Math1-creER*^*T2*^::*Smarca4*^*fl/fl*^ mice, pups received a single dose of 0.4 mg tamoxifen dissolved in corn oil by intraperitoneal injection at postnatal day 3 (P3).

### Lentivirus production

A lentiviral plasmid driving overexpression of MYC was generated by cloning the murine *Myc* gene from a previously described MSCV-MYC-IRES-RFP construct [32] into a self-designed lentiviral expression vector backbone (pLV-CMV-IRES-GFP) ordered from VectorBuilder, IL, USA. Production and titration of second generation lentiviral particles was performed by transfection of HEK293T cells as previously described [57]. Viral particles were concentrated by ultracentrifugation and stored at -80 °C before transduction.

### Culture of granule cell precursors (GCPs)

Primary murine GCPs were isolated from *Math1-creER*^*T2*^::*Smarca4*^*fl/fl*^ or *Smarca4*^*fl/fl*^ pups at P7 or P8 as previously described [42]. Lentiviral transduction of GCPs with MYC (pLV-CMV-MYC-IRES-GFP) or Mock (pLV-CMV-IRES-GFP) constructs was performed 4 h after isolation with addition of protamine sulfate (8 µg/ml) and centrifugation at 2,000 rpm for 1 h. Medium was changed the next morning with concurrent exchange of FCS-supplemented medium to serum-free medium containing 3 µg/mL SHH protein. Bromodeoxyuridine (BrdU) was added to the cells at a concentration of 25 µg/mL for 2 h before fixation of cells. For orthotopic transplantation, transduced GCPs were dissociated with Accutase 24 h after isolation and were washed and resuspended in a solution of 3:1 medium and Matrigel on ice.

### Stereotactic transplantations

During stereotactic transplantations, recipient mice (6-week-old *CD1*^*nu/nu*^) were anesthetized by isoflurane inhalation. They additionally received analgesia by subcutaneous injections of carprofen (6 mg/kg) before transplantation and on the day after. For the procedure, mice were placed in a stereotactic frame (David Kopf Instruments, CA, USA) on a heating pad, and eye ointment was applied to avoid dehydration. Local anesthesia (2% lidocaine) was applied before performing a skin incision and puncturing the skull for injection. A total of 1.5 × 10^6^ cells were injected using a Hamilton syringe (World Precision Instruments, FL, USA) at coordinates x: +1 mm, y: -1 mm, and z: -2 mm from the lambda suture at 30° from the skull surface. Mice were monitored daily for any sign of tumor development within the following six months.

### Immunohistochemistry (IHC)

For histological examination of brains, tissue was fixed in 4% formaldehyde for at least 12 h. The tissue was dehydrated, embedded in paraffin, and sectioned at 2 μm according to standard protocols. Hematoxylin and eosin (HE) stainings were applied according to standard protocols. 3,3’-Diaminobenzidine (DAB) stainings were performed on a Ventana Benchmark system using the ultraView or OptiView DAB detection kit (all Roche Diagnostics, Basel, CH). The following antibodies were used: Cleaved Caspase-3 (CC-3): Cell Signaling #9664, RRID:AB_2070042 (1:100); GFP: Abcam #ab290, RRID:AB_303395 (1:500); Ki67: Abcam #ab15580, RRID:AB_443209 (1:100); MYC: Zeta Corporation #Z2734RL (1:25); Nestin: Abcam #ab221660, RRID:AB_2909415 (1:2000); NeuN: Merck #MAB377, RRID:AB_2298772 (1:50); OLIG2: Merck #AB9610, RRID:AB_570666 (1:200); SMARCA4: Abcam #ab110641, RRID:AB_10861578 (1:25); and SOX2: Abcam #92,494, RRID:AB_10585428 (1:200).

### Immunofluorescence (IF) stainings

IF stainings of formalin-fixed paraffin-embedded (FFPE) tissue were performed manually after deparaffinization and antigen retrieval with citrate buffer. For IF staining of GCPs in vitro, cells were fixed with 4% formaldehyde for 10 min. In case of BrdU stainings, acidic pre-treatment (4 N HCl and 0.1 M sodium borate for 10 min each) was performed before blocking with 10% NGS in 0.3% Triton X-100. The following primary antibodies were used for incubation at 4 °C over night: BrdU: Invitrogen #B35128, RRID:AB_2536432 (1:100); MYC: Cell Signaling #5605, RRID:AB_1903938 (1:800); GFP (mouse): Invitrogen #A11120, RRID:AB_221568 (1:100); GFP (rabbit): Invitrogen #A11122, RRID:AB_221569 (1:100); and SMARCA4: Abcam #ab110641, RRID:AB_10861578 (1:25). Secondary antibodies (1:500) and DAPI (1 µg/ml) were added for 1 h at room temperature on the next day: anti-mouse Alexa 488: Cell Signaling Technology #4408S, anti-mouse Alexa 555: Cell Signaling Technology #4409S, anti-rabbit Alexa 488: Cell Signaling Technology #4412S, and anti-rabbit Alexa 546: Invitrogen #A11035.

### Image quantifications

IF stainings of GCPs were quantified automatically using the *Automatic Measurement* tool of the NIS-Elements (AR 5.11.03) software. The threshold for fluorescence intensity and cell size was adjusted separately for each fluorescence channel and was applied to all samples to retrieve cell counts. At least three representative images were analyzed for each sample and staining. DAB stainings of tumors (MYC and GFP) were quantified with Image J (v 1.48a). All evaluated stainings were performed with the automated Ventana system and within the same run to ensure comparability of detected signals. Five pictures were taken from different areas within the tumor, DAB color deconvolution was applied, and resulting images (Color 2) were converted into 8-bit format. Masks with the following black/white thresholds were applied before measuring the corresponding area fraction: high signal: 0-125, medium signal: 125–150, low signal: 150–175, no signal: 175–255.

### Western blot

For Western blotting, 30 µg of protein per sample were separated by SDS-PAGE (4–10% gradient) and were transferred onto a nitrocellulose membrane. After blocking with 5% milk powder in TBS-Tween, the membrane was incubated with the primary antibody at 4 °C overnight. The following antibodies were used: β-tubulin: Sigma-Aldrich #T4026, RRID:AB_477577 (1:500); GAPDH: GeneTex #100,118, RRID:AB_1080976; MYC: Cell Signaling #5605, RRID:AB_1903938 (1:500); and SMARCA4: Abcam #ab110641, RRID:AB_10861578 (1:10,000). After washing, the secondary horse-radish peroxidase (HRP) coupled antibody was applied for 1 h at room temperature: Goat-anti-mouse-HRP: Dako #P0447 (1:10,000) or Goat-anti-rabbit-HRP: Dako #P0448 (1:10,000). The Clarity Western ECL Substrate (Bio-Rad Laboratories Inc, CA, USA) was used for detection on X-ray films.

### RNA sequencing analysis

RNA Isolation from FFPE tissue was performed using the Maxwell RSC RNA FFPE kit (Promega Corporation, WI, USA). Prior to sequencing, RNA concentration and integrity was determined on an RNA 6000 Nano Chip on the Agilent 2100 Bioanalyzer system (Agilent Technologies, CA, USA). At least 100 ng total RNA per sample were used for sequencing. Ribosomal RNA was depleted with the RiboCop Human/Mouse/Rat V2 kit before library preparation with the CORALL Total RNA-seq V2 kit (both Lexogen GmbH, Vienna, AT). Pooled libraries were sequenced on a NextSeq500 sequencing system (Illumina, CA, USA) by 1 × 75 bp single-end sequencing for 75 cycles, generating at least 30 Mio reads per sample.

Raw fastq files of mouse samples were processed in usegalaxy.eu [1]. Low quality reads were detected using *FastQC* (Galaxy Version 0.73 + galaxy0), and reads with average quality < 20 were trimmed with *Trimmomatic* (Galaxy Version 0.38.1). Reads were aligned to the GRCm39 (mm39) mouse reference genome using *STAR aligner* (Galaxy Version 2.7.8a + galaxy1). Gene expression was quantified with *featureCounts* (Galaxy Version 2.0.1 + galaxy2), and VST-normalized files were generated by *DEseq2* (Galaxy Version 2.11.40.7 + galaxy2). Further processing of data was performed with R (4.2.1).

Differential gene expression analysis between mouse samples was performed using *limma* (3.52.2) [54]. Genes orthologous to humans were used for volcano plots generated with *ggplot2* (3.4.1) with genes considered differentially expressed if LogFC ≥ 2.5 and False Discovery Rate (FDR) adjusted p ≤ 0.01. For gene set enrichment analysis, all mouse genes with LogFC ≥ 1.5 and FDR adjusted p ≤ 0.01 were considered using multiple packages from *clusterProfiler* (4.4.4) visualized with in-built *clusterProfiler* plots.

Human gene expression data were obtained from a previously published pediatric brain tumor cohort (Sturm et al. 2016 [61]; GSE73038). To compare mouse and human gene expression data, 14,151 orthologous genes between both datasets were used, and data were batch corrected for species differences using an in-house pipeline. The previously identified 14,151 orthologous genes were used for differential gene expression analysis between human tumor subtypes using *limma* (3.52.2) [54]. The 6,000 most differentially expressed genes (or 5,000 for MB only) were selected using Benjamini-Hochberg correction for multiple testing and sorting by F-statistic. Visualizations were performed using RStudio packages *umap* (0.2.9.0) [41] and *Complex Heatmap* (2.12.1) [18] using Euclidian distance and Ward.D2 linkage for clustering. For the distance plots, Euclidean distance was measured (*Stats* 4.1.2 package), and plots were generated with *Complex Heatmap*.

### DNA methylation analysis

DNA from frozen tumor biopsies (tumors 3 + 4) was isolated using the NucleoSpin Tissue kit (Macherey-Nagel, Düren, DE), whereas DNA isolation from FFPE tissue (tumor 1) was performed using the Maxwell RSC DNA FFPE kit (Promega Corporation). At least 150 ng of total DNA were used for bisulfite conversion with the EZ DNA Methylation kit (Zymo Research, CA, USA). Then, samples were analyzed on the Infinium Mouse Methylation BeadChip array covering > 285,000 CpG sites within the mouse genome on an iScan array scanner (both Illumina). Human tumor samples were analyzed on the MethylationEPIC 850k BeadChip array (Illumina). The use of biopsy-specimens for research upon anonymization was always in accordance with local ethical standards and regulations at the University Medical Center Hamburg-Eppendorf.

Data processing and analysis was performed with R (4.1.2). For preprocessing of raw data and extraction of beta values, the *Minfi* package [3] was used for human data, whereas the *SeSAMe* package [70] was used for mouse data. Then, quantile normalization of data was performed. For a comparison of murine samples to human brain tumor DNA methylation profiles, previously published data by Capper et al. [9] and Sharma et al. [59] were combined with data generated in-house (in total n = 228). Within the human dataset including all brain tumor entities, the 15,000 most differentially methylated CpG sites were identified. Out of these, 491 CpGs that are orthologous between the human and mouse genome were chosen for further analysis. Human and mouse datasets were combined and again, quantile normalization was performed. UMAPs [41] as well as hierarchically clustered heatmaps (*Complex Heatmap* package [18]) were generated based on the differential methylation of the previously chosen 491 CpGs. For the generation of distance plots, Pearson correlation (*Stats* 4.1.2 package) was applied, and plots were generated with the *Complex Heatmap* package.

### Statistical analysis

All statistical analysis was performed using the GraphPad Prism (9.4.1) or R (4.1.2) software. The statistical tests applied to the data shown are stated in the respective figure legends. For each comparison, at least n = 3 samples per group were used and/or n = 3 independent experiments were conducted. P-values were corrected for multiple testing. All graphs depict mean values +/- standard deviation.

## Results

### Loss of SMARCA4 or MYC overexpression does not increase proliferation of granule cell precursors (GCPs) in vitro

**Fig. 1:**
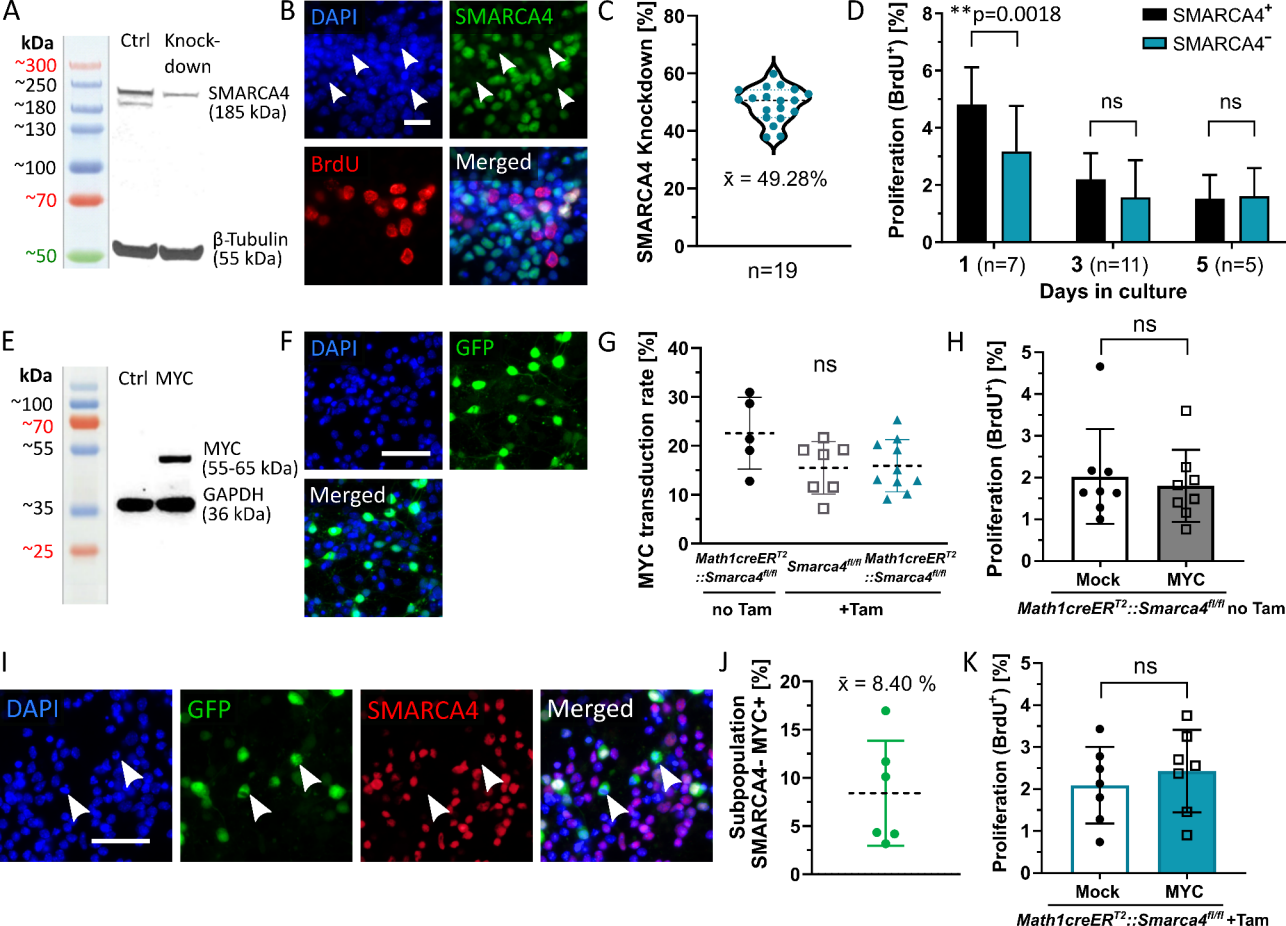
**Loss of SMARCA4 or MYC overexpression does not increase proliferation of GCPs in vitro**. (**A**) Tamoxifen-induced knockdown of SMARCA4 is evident in Western Blot of P7/8 *Math1creER*^*T2*^::*Smarca4*^*fl/fl*^ GCPs compared to controls (*Smarca4*^*fl/fl*^) after tamoxifen injection at P3. Two SMARCA4 bands are detected as seen in previously published studies [19, 46]. (**B**) IF staining of knockdown GCPs at day 3 in culture shows loss of SMARCA4 protein and proliferation indicated by BrdU incorporation. White arrowheads mark SMARCA4-negative areas. (**C**) Evaluation of SMARCA4 knockdown in IF on day 3 in culture of 19 independent GCP cultures. (**D**) Proliferation as measured by BrdU incorporation in IF on day 1, 3, and 5 in culture, separately counted for SMARCA4-positive and -negative GCPs in knockdown cultures. Two-tailed paired t-tests were applied. (**E**) MYC expression is evident in Western Blot of wild-type P7/8 GCPs 72 h after transduction. (**F**) IF staining shows GFP signal 72 h after transduction of GCPs. (**G**) MYC transduction rates were evaluated in IF stainings of GCPs 72 h after transduction. The three groups include GCPs without tamoxifen (Tam) induction and GCPs of cre-negative (*Smarca4*^*fl/fl*^) and cre-positive (*Math1-creER*^*T2*^::*Smarca4*^*fl/fl*^) genotype after tamoxifen induction at P3. Tukey’s multiple comparisons test was applied. (**H**) Overall proliferation as measured by BrdU incorporation in IF of *Math1creER*^*T2*^::*Smarca4*^*fl/fl*^ GCPs without tamoxifen induction 72 h after transduction with Mock or MYC constructs. Paired two-tailed t-test was applied. (**I**, **J**) IF staining of tamoxifen-induced *Math1creER*^*T2*^::*Smarca4*^*fl/fl*^ GCPs 72 h after transduction with MYC virus. The subpopulation with SMARCA4 protein loss and GFP signal (white arrowheads) constitutes around 8.4% of the whole cell culture. (**K**) Overall proliferation of tamoxifen-induced *Math1creER*^*T2*^::*Smarca4*^*fl/fl*^ GCPs 72 h after transduction with Mock or MYC constructs. Paired two-tailed t-test was applied. Scale bar in B corresponds to 20 μm, scale bars in F + I correspond to 50 μm

In a first step, we investigated the influence of both *Smarca4* and *Myc* alterations on cell behavior in vitro. To induce a loss of SMARCA4 in GCPs, *Math1-creER*^*T2*^::*Smarca4*^*fl/fl*^ mice received a single dose of tamoxifen at P3, and GCPs were isolated from the cerebella when pups reached an age of 7–8 days. Successful knockdown of SMARCA4 was detected in around 50% of cells as shown in Western Blot and IF stainings (Fig. 1A-C). Proliferation was significantly decreased in SMARCA4-negative cells at day 1 in culture, while no significant difference in proliferation was observed at day 3 or 5 in culture (Fig. 1D). Next, we analyzed the effect of MYC overexpression in GCPs by transduction with a lentiviral MYC-GFP construct. Successful transduction was validated by the presence of MYC protein in Western Blot and by positive GFP IF stainings with mean transduction rates ranging between 15.5 and 22.6% (Fig. 1E-G). Overall proliferation of non-induced *Math1-creER*^*T2*^::*Smarca4*^*fl/fl*^ GCPs after transduction with MYC virus showed no difference compared to proliferation of cells transduced with a Mock-GFP construct (Fig. 1H). Subsequently, we combined both SMARCA4 loss and MYC overexpression by transducing tamoxifen-induced *Math1creER*^*T2*^::*Smarca4*^*fl/fl*^ GCPs. As shown in Fig. 1I-J, the subpopulation of SMARCA4-deficient GFP-positive GCPs constituted around 8.4% of the whole cell culture. Again, overall proliferation was not significantly increased after MYC transduction (Fig. 1K). However, proliferation of the SMARCA4-deficient and successfully transduced subpopulation could not be analyzed separately since acidic pre-treatment required for BrdU stainings destroys GFP epitopes [7].

### Loss of SMARCA4 and MYC overexpression cooperate to drive brain tumor formation in vivo

**Fig. 2:**
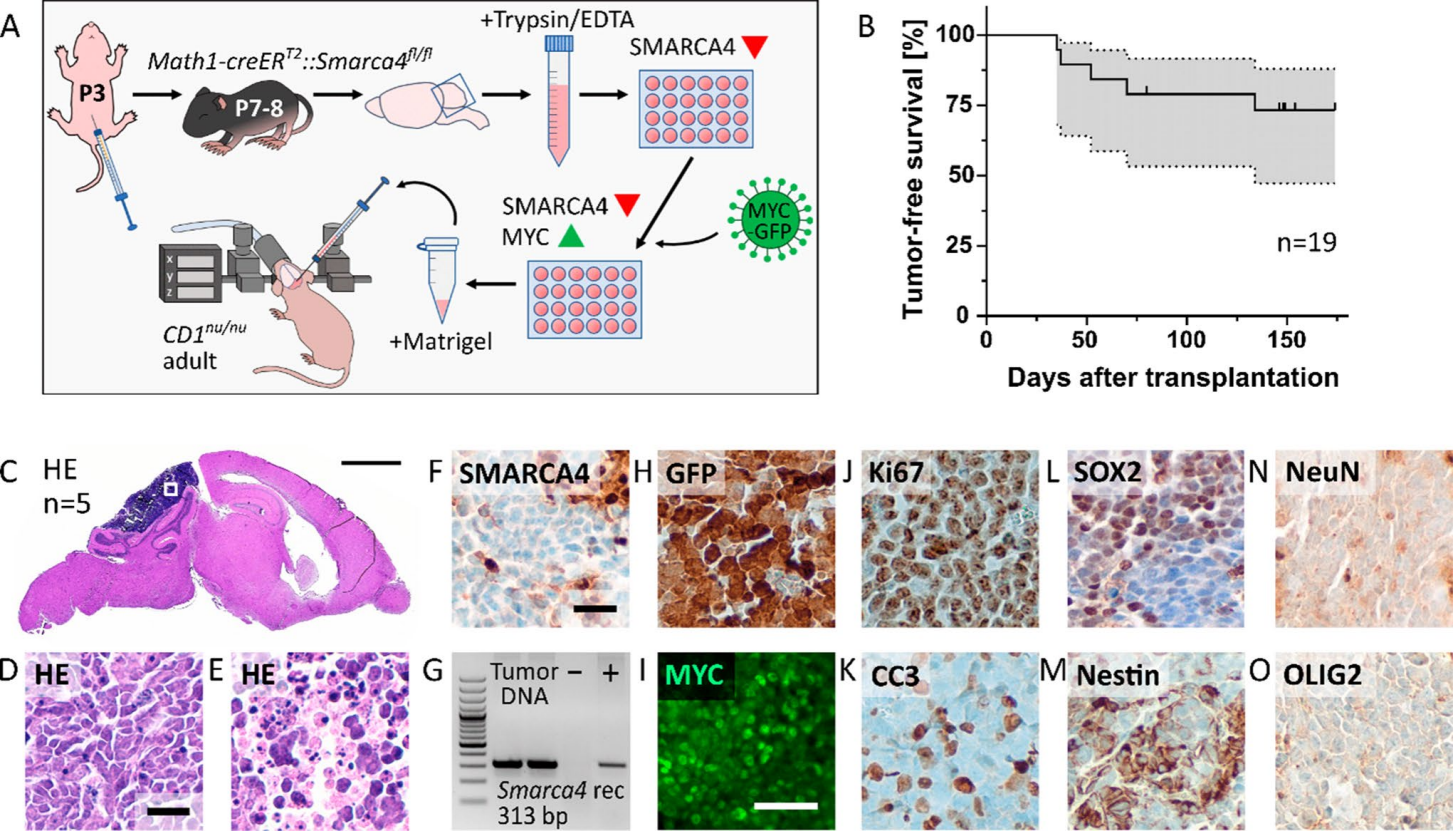
**Loss of SMARCA4 and MYC overexpression cooperate to drive brain tumor formation in vivo**. (**A**) Schematic overview of the cell culture and transplantation protocol for the generation of SMARCA4-deficient MYC-overexpressing tumors. (**B**) Tumor-free survival of transplanted *CD1*^*nu/nu*^ mice; grey area represents the 95% confidence interval. Censored mouse at day 80 had to be sacrificed due to illness unrelated to tumor development. (**C**) Representative HE staining of tumors in the brains of n = 5 transplanted mice in sagittal brain section. (**D**,**E**) High-power HE stainings of distinct areas within the tumors showing (**D**) anaplastic or (**E**) apoptotic features. (**F**) Tumors show complete loss of SMARCA4 in IHC interspersed with SMARCA4-positive blood vessels. (**G**) PCR using DNA isolated from tumor biopsies confirms *Smarca4* recombination on a genetic level. (**H**-**I**) Tumors stain positive for (**H**) GFP and (**I**) MYC, confirming transduction with the MYC-GFP construct. (**J**) Tumors are highly proliferative as indicated by Ki67 stainings; (**K**) with a high degree of apoptosis according to Cleaved Caspase-3 (CC3) signals. (**L-O**) Tumors show scattered expression of (**L**) SOX2 and (**M**) Nestin but no signal for (**N**) NeuN or (**O**) OLIG2. Scale bar corresponds to 2 mm in C, to 25 μm in D + F (also applicable to **E**, **H**, **J-O**), and to 50 μm in I

In a next step, we transplanted altered GCPs into immunodeficient *CD1*^*nu/nu*^ mice to further explore their tumorigenic potential in vivo. For this purpose, SMARCA4 knockdown GCPs were isolated from induced *Math1creER*^*T2*^::*Smarca4*^*fl/fl*^ mice and were transduced with a lentiviral MYC construct as described above. On the next day, GCPs were dissociated and transplanted into the cerebella of *CD1*^*nu/nu*^ mice without pre-sorting for recombined or transduced cells (Fig. 2A). Within a cohort of 19 transplanted mice, five mice developed a tumor in the cerebellum, presenting with neurological symptoms earliest four weeks and latest five months after transplantation (Fig. 2B). Histologically, tumors presented as a cell dense mass in HE stainings, with regions showing anaplastic features as well as apoptotic areas, consistent with large cell/anaplastic (LCA) histology frequently detected in MYC driven Group 3 MB (Fig. 2C-E) [13, 30]. IHC stainings revealed a loss of SMARCA4 in all tumor cells (Fig. 2F). The presence of recombined *Smarca4* in tumor biopsies was also verified by PCR, which confirmed that the loss of SMARCA4 was caused by genetic recombination (Fig. 2G). Furthermore, tumors stained positive for both GFP and MYC, thereby validating successful transduction with the MYC-GFP construct (Fig. 2H + I). Tumors were highly proliferative according to Ki67 signals and displayed a high degree of apoptosis as indicated by Cleaved Caspase-3 (CC3) staining (Fig. 2J + K). Staining for neural markers revealed scattered expression of SOX2 and Nestin, whereas no signal for NeuN or OLIG2 was detected (Fig. 2L-O). Altogether, these results affirmed the origin of detected tumors in the subpopulation (8.4%) of GCPs harboring both recombined *Smarca4* and overexpressed MYC and showed proliferative capacity as well as undifferentiated nature of tumors.

Intratumoral heterogeneity of *MYC* amplification within Group 3 MB has been described as an important factor in metastasis and therapy resistance [51]. Therefore, we analyzed levels of MYC expression in different regions of our tumors, which revealed striking heterogeneity in between but also within samples (Additional File 1, Fig. S1A-C). All tumors contained areas with varying degrees of MYC signal including cells without any MYC signal. In contrast, GFP signals were uniformly high in all tumors, suggesting regulation of MYC expression independent from successful transduction with the MYC-GFP construct (Additional File 1, Fig. S1D-F).

Moreover, we examined brains and spines for leptomeningeal dissemination, which is detected in around 40% of human Group 3 MB and has also been recapitulated in other MYC-driven medulloblastoma models [33, 38]. In our model, we observed leptomeningeal spread within the brain in four out of five tumor-bearing mice, affecting the cerebral cortex, the midbrain, and the brain stem (Additional File 1, Fig. S1G-J). However, we did not detect any dissemination in the spines of affected mice.

### Differential gene expression in MYC/SMARCA4 tumors

**Fig. 3:**
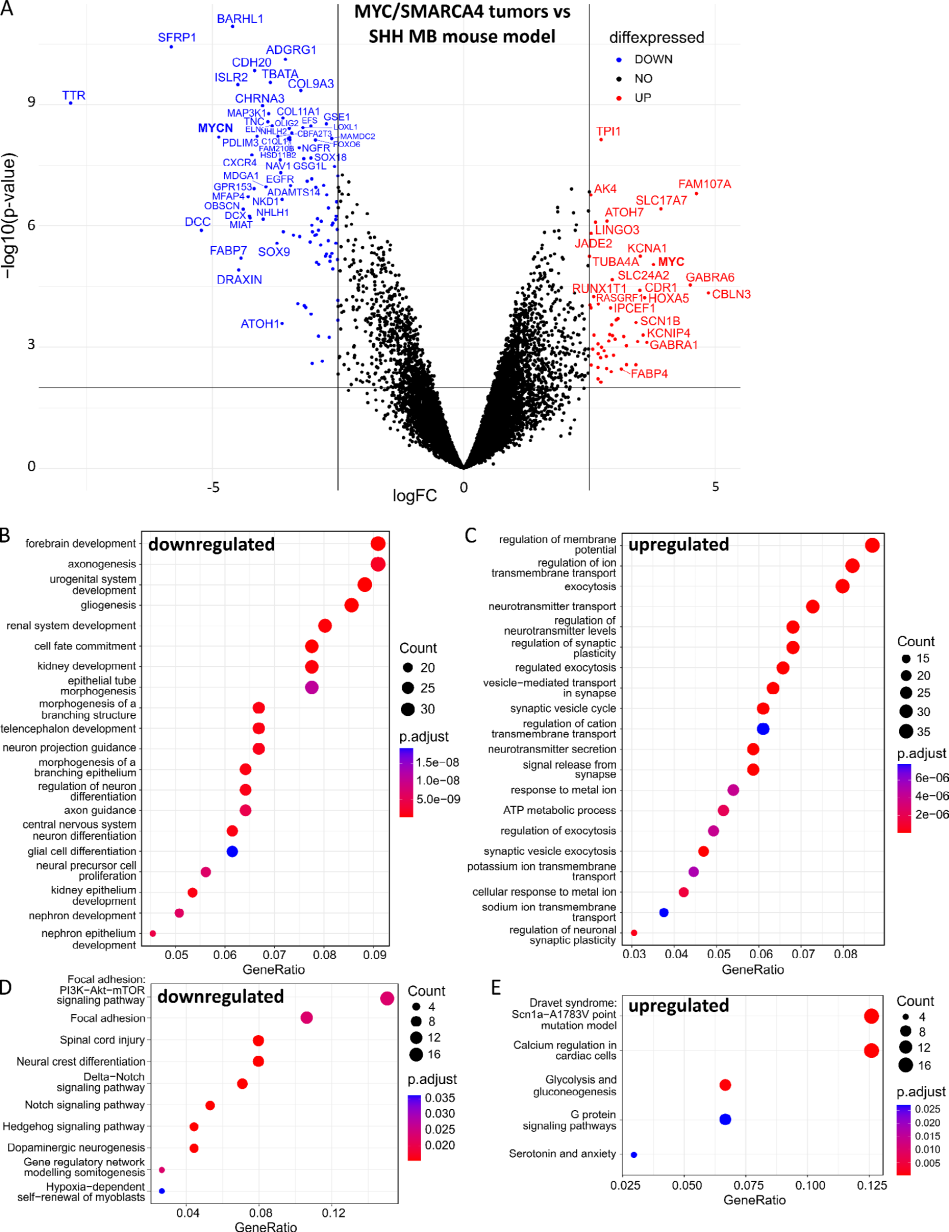
**Differential gene expression of MYC/SMARCA4 tumors compared to an established mouse SHH MB mouse model**. (**A**) Volcano plot depicting differential gene expression between our MYC/SMARCA4 tumor model (n = 4) and the *Math1-cre::Smo*^*fl/wt*^ SHH MB mouse model (n = 3) as assessed by RNA sequencing analysis. Only genes orthologous in mice and humans were visualized, and differential expression with logFC ≥ 2.5 and p ≤ 0.01 was considered significant (blue/red coloring) after Benjamini-Hochberg correction. A detailed list of differentially expressed genes is included in Additional File 2, Table S2. (**B**,**C**) Gene set enrichment analysis was performed based on significantly differentially expressed genes considering all mouse genes with logFC ≥ 1.5 and p ≤ 0.01. (**D**,**E**) Deregulated wiki pathways considering differentially expressed genes across all mouse genes with logFC ≥ 1.5 and p ≤ 0.01

To characterize MYC/SMARCA4 tumors on a molecular level, we performed RNA sequencing using FFPE biopsy punches of four mouse tumors. As a control, we simultaneously sequenced FFPE-derived RNA of a previously established SHH MB mouse model (*Math1cre::Smo*^*fl/wt*^ [58]) and of *Math1creER*^*T2*^::*Smarca4*^*fl/fl*^ P7 whole cerebella. The comparison of MYC/SMARCA4 tumors to *Math1creER*^*T2*^::*Smarca4*^*fl/fl*^ cerebella revealed *Myc* as the most significantly upregulated gene in our model (Additional File 1, Fig. S2A; Additional File 2, Table S1). Gene set enrichment analysis revealed downregulation of terms associated with neuronal development and differentiation, while upregulated terms were mainly associated with ribosome biogenesis and ribosomal RNA (rRNA) synthesis and processing, a characteristic hallmark for MYC-driven cancers (Additional File 1, Fig. S2B,C) [66]. Comparison of gene expression profiles of MYC/SMARCA4 tumors to the established SHH MB mouse model again confirmed upregulation of *Myc*, while *MycN* as a target of SHH signaling was significantly downregulated (Fig. 3A; Additional File 2, Table S2). Other downregulated genes included *Atoh1* and *Barhl1*, both markers for granule cells, of which low levels of *BARHL1* have been associated with a less favorable prognosis in MB [50]. On the other hand, *Hoxa5* and *Fabp4*, both associated with increased malignancy in gliomas, were upregulated in MYC/SMARCA4 tumors [11, 21]. Gene set enrichment analysis revealed downregulated GO terms mostly linked to neuronal development (Fig. 3B). Meanwhile, terms associated with transmembrane transport and synaptic signaling were upregulated in our model (Fig. 3C). Pathway analysis confirmed the downregulation of SHH signaling but also reduction of Notch and PI3K-Akt-mTOR signaling, whereas glycolysis/gluconeogenesis as well as G protein signaling pathways were upregulated in MYC/SMARCA4 tumors (Fig. 3D + E).

### MYC/SMARCA4 tumors show molecular resemblance to human Group 3 MB

**Fig. 4:**
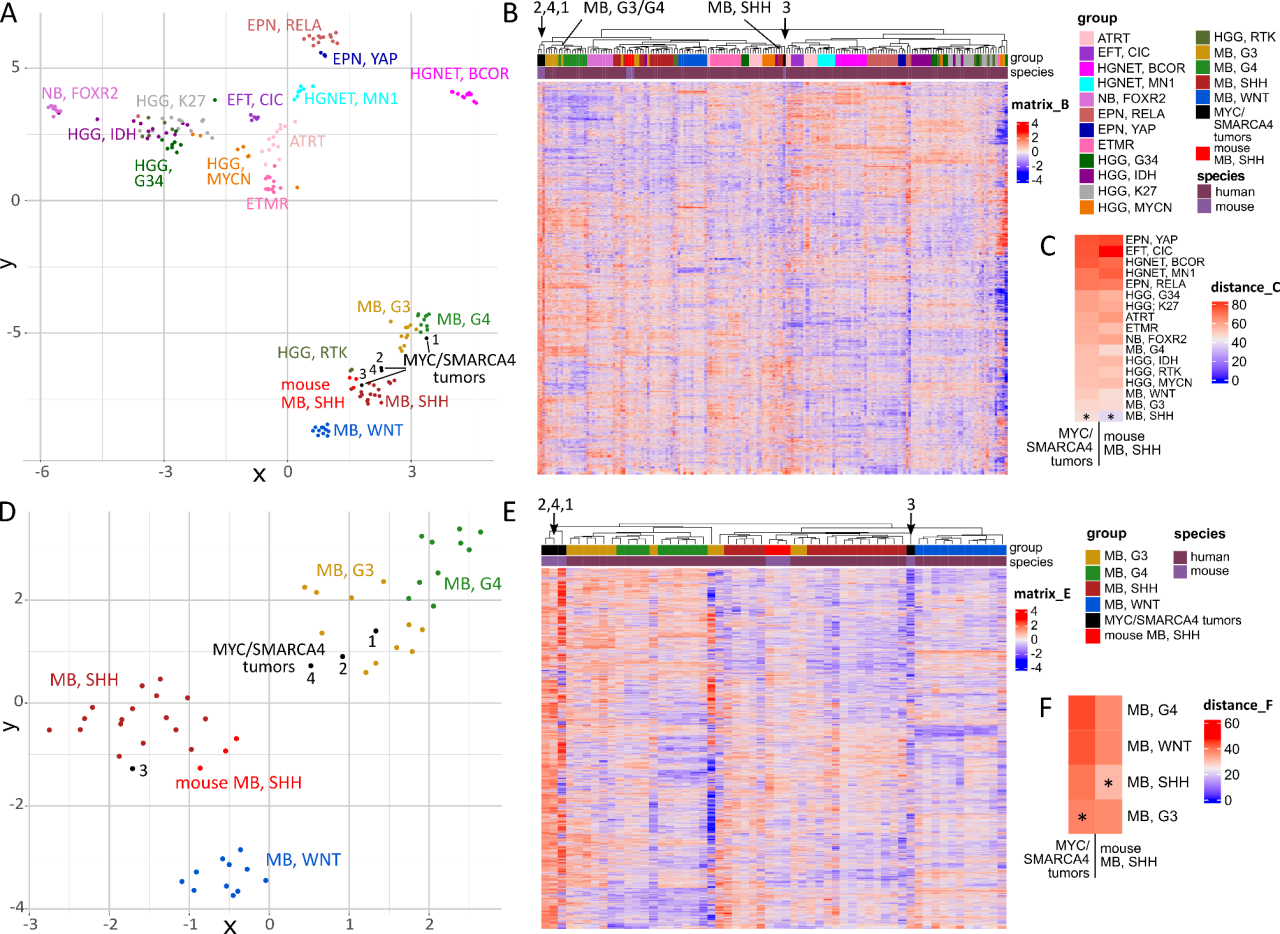
**MYC/SMARCA4 tumors show similarities to Group 3 MB in gene expression analysis**. (**A**) UMAP clustering of mouse tumors profiled by RNA sequencing and published expression data of pediatric brain tumors (Sturm et al. 2016 [61]). Out of the 14,151 orthologous genes identified between both datasets, the 6,000 most differentially expressed genes within the human dataset were used for clustering. Mouse SHH MB show resemblance to their human counterpart, whereas MYC/SMARCA4 tumors display similarity to both SHH MB and Group 3/4 MB. (**B**) Hierarchical clustering according to differentially expressed genes shows proximity of MYC/SMARCA4 tumors to the Group 3/4 MB cluster for three samples, whereas tumor 3 clusters with a subset of SHH MB (black arrows). (**C**) Distance plot shows closest resemblance of both mouse tumor models to SHH MB. Asterisks mark shortest distance. (**D**) UMAP clustering of mouse tumors and human MB subgroups only (Sturm et al. 2016) according to the 5,000 most differentially expressed genes within the human MB dataset out of 14,151 orthologous genes. MYC/SMARCA4 tumors appear closest to Group 3 MB. (**E**) Hierarchical clustering confirms proximity of MYC/SMARCA4 tumors to the Group 3/4 MB cluster with exception of tumor 3 (black arrows). (**F**) Distance plot shows closest resemblance of MYC/SMARCA4 tumors to Group 3 MB. EFT, CIC = Ewing sarcoma family tumor with *CIC* alteration; HGNET, BCOR = High-grade neuroepithelial tumor with *BCOR* alteration; NB, FOXR2 = Neuroblastoma with *FOXR2* activation; EPN, RELA = Ependymoma with *RELA* fusion; EPN, YAP = Ependymoma with *YAP* fusion; ETMR = Embryonal tumor with multilayered rosettes; HGG, G34 = *H3F3A* G34 mutant high-grade glioma; HGG, IDH = *IDH* mutant high-grade glioma; HGG, K27 = *H3F3A* K27 mutant diffuse midline glioma; HGG, MYCN = *MYCN*-amplified high-grade glioma; HGG, RTK = *IDH/H3F3A* wild-type high-grade glioma of the receptor tyrosine kinase (RTK) subtype; MB, G3 = MB, Group 3; MB, G4 = MB, Group 4

In a next step, we integrated our RNA sequencing data with previously published gene expression data to test comparability of our murine tumors to human brain tumors. An integration with a data set comprising several pediatric brain tumor entities (Sturm et al. 2016 [61]) revealed resemblance of our model to human MB in both UMAP and Euclidian clustering (Fig. 4A-B). While mouse SHH MB serving as a validation displayed unambiguous proximity to human SHH MB, MYC/SMARCA4 tumors showed similarity to both SHH MB and Group 3/4 MB in both approaches. A distance plot analysis considering mean values for each subgroup indicated closest proximity of both mouse SHH MB and our MYC/SMARCA4 tumors to human SHH MB (Fig. 4C). Based on these results, we further evaluated the similarity to specific MB subgroups by comparing our mouse model exclusively to MB samples. Within the human MB cohort, we again performed gene expression analysis to identify the most differentially expressed genes between MB subgroups. An integration of our mouse data resulted in closest similarity of MYC/SMARCA4 tumors to Group 3 MB in both UMAP and Euclidian clustering, whereas mouse SHH MB reliably clustered with human SHH MB (Fig. 4DE). In both approaches, tumor 3 formed an exception by clustering closely with SHH MB. However, we did not detect apparent differences to the other three samples in histological appearance and levels of MYC or SMARCA4 in this tumor. Distance plot analysis further confirmed closest proximity of MYC/SMARCA4 tumors to Group 3 MB (Fig. 4F).

**Fig. 5:**
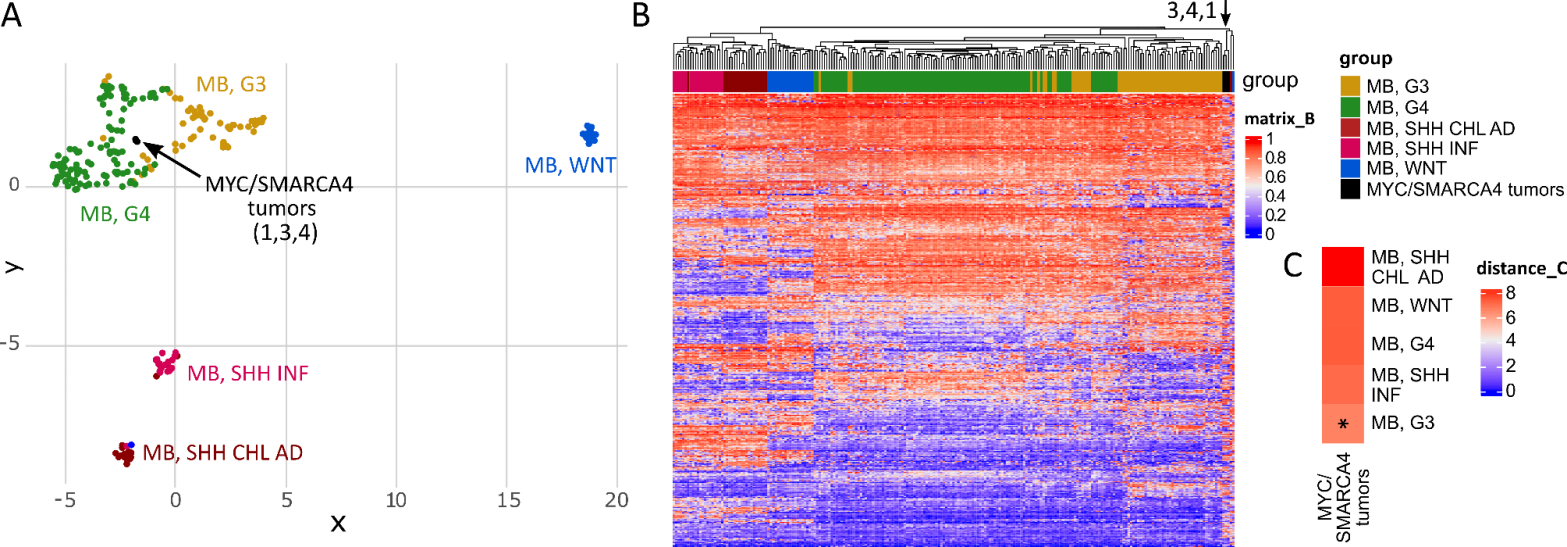
**MYC/SMARCA4 tumors show similarities to Group 3/4 MB in DNA methylation analysis**. (**A**) UMAP clustering according to DNA methylation of mouse tumors (Mouse Methylation BeadChip) and human MB (Capper et al. 2018 [9], Sharma et al. 2019 [59], and in-house analyzed samples, n = 228) using 491 orthologous CpG sites out of the 15,000 most differentially methylated CpG sites within the human dataset. Mouse MYC/SMARCA4 tumors (n = 3) show most similarity to MB, Group 3/4. (**B**) Heatmap clustering according to DNA methylation of the same samples and CpG sites similarly shows proximity of the MYC/SMARCA4 tumors to MB, Group 3/4 (black arrow). (**C**) Distance Plot using the mean methylation values summarized for every subgroup shows lowest distance of MYC/SMARCA4 tumors to MB, Group 3. MB, G3 = MB, Group 3; MB, G4 = MB, Group 4; MB, SHH CHL AD = Medulloblastoma SHH-activated (children and adults); MB, SHH INF = Medulloblastoma SHH-activated (infants)

Human brain tumors and biologically relevant tumor subgroups can be reliably classified according to their DNA methylation profile [9]. Therefore, we additionally isolated DNA of three mouse tumors (tumors 1, 3, and 4 from RNA sequencing analysis) and performed global DNA methylation analysis using the Mouse Methylation Bead Chip. These data were integrated with a human MB dataset comprising in-house analyzed samples and previously published cohorts [9, 59]. UMAP and Euclidian clustering according to differential methylation of 491 orthologous CpG sites showed good separation of human MB subgroups, with MYC/SMARCA4 tumors clustering in close proximity to Group 3/4 MB (Fig. 5A-B). A distance plot confirmed highest resemblance of MYC/SMARCA4 tumors to Group 3 MB (Fig. 5C).

## Discussion

In this study, we successfully generated SMARCA4-deficient tumors in mice resembling human Group 3 MB both histologically and molecularly. Although SMARCA4 loss or MYC overexpression did not increase proliferation of GCPs in vitro, the combination of both alterations induced tumor formation after orthotopic transplantation in vivo. An important role of altered SMARCA4 in MB development was suspected before since the overexpression of SMARCA4 wild-type represses tumor development in an OTX2/MYC Group 3 MB mouse model [5]. Our study now confirmed these assumptions by showing a selection for SMARCA4-deficient cells in all detected MYC/SMARCA4 tumors.

On its own, a loss of SMARCA4 in GCPs does not harbor tumorigenic potential as indicated by decreased proliferation of SMARCA4-deficient GCPs in vitro. This observation could be attributed to the previously described failure of SMARCA4-deficient GCPs to respond to SHH protein, which is added to cell cultures as a mitogen [69]. Moreover, we have shown before that a postnatally induced loss of SMARCA4 in *Math1-creER*^*T2*^::*Smarca4*^*fl/fl*^ mice delays migration of GCPs to the internal granular layer in vivo but does not affect the cerebellar phenotype seen later in development [26]. Similarly, overexpression of MYC alone did not increase proliferation of GCPs in vitro. In contrast, Pei et al. have shown higher proliferation and increased ability to form neurospheres after transducing cerebellar stem cells with a stabilized MYC^T58A^ construct [47]. Lentiviral transduction of SOX2-positive cerebellar progenitors with MYC^T58A^ is even sufficient to drive formation of Group 3-like MB in mice [64]. However, the choice of a wild-type MYC construct in our study could play a crucial role. Kawauchi et al. did not detect development of MB after overexpression of wild-type MYC alone by *in utero* electroporation [33]. Moreover, Swartling et al. have shown that overexpression of stabilized MYCN^T58A^ in neural stem cells results in the development of brain tumors, while overexpression of wild-type MYCN does not [63]. Consequently, aberrant chromatin remodeling by the loss of SMARCA4 in our model might cause stabilization of wild-type MYC required for the development of tumors.

The fact that MYC/SMARCA4 tumors did not only show high resemblance to the transcriptome of Group 3 MB but also displayed similarities to SHH MB could be attributed to the cellular origin of our tumors. SHH MB are derived from GCPs as previously demonstrated in several mouse models and confirmed by comparisons to single-cell RNA sequencing data of murine and human cell populations [4, 58, 60, 67]. In our model, we specifically targeted Math1-positive GCPs by tamoxifen-induced *Smarca4* recombination at P3. GCPs are among many other neural progenitor populations that have been used before to model Group 3 MB in mice [33, 38, 64]. This fits to the fact that the exact cellular origin of Group 3 MB cannot be clearly assigned to a single murine cell population in the brain [67]. Indeed, recently published work provides evidence for both Group 3 and 4 MB originating from a distinct cell population in the subventricular zone of the human rhombic lip that does not exist in mice [22, 35, 60]. This divergence from previously used cells of origin should be considered in future attempts at modeling Group 3 MB in mice.

Nevertheless, SMARCA4-deficient MB mouse models could provide a valuable platform to explore targeted therapeutic options for affected patients. For now, the limited penetrance of our tumor model restricts its suitability for such studies. Pre-sorting for successfully transduced cells before transplantation could increase the fraction of MYC-overexpressing SMARCA4-deficient cells within the injected mixture, possibly also enhancing engraftment. However, this would also entail one more day of in vitro culture before transplantation for the GFP signal to be detectable. Consequently, fragile SMARCA4-deficient GCP cultures might show reduced viability and proliferative capacity by then. Alternative approaches include the introduction of an additional SMARCA4 deficiency in a recently developed transgenic MYC driven MB mouse model or the use of other promoters such as *Blbp-cre* or *GFAP-cre* to drive earlier deletion of SMARCA4 [38].

In comparing gene expression profiles of our MYC/SMARCA4 tumors to an established SHH MB model, we identified upregulation of G protein signaling and glucose metabolism in our tumor model. Tao et al. have previously shown altered glucose metabolic pathways in a MYC driven MB mouse model and were successful in treating tumor cells with specific inhibitors of upregulated lactate dehydrogenase A [64]. Furthermore, several studies have suggested histone deacetylase (HDAC) inhibitors for treating MYC-driven Group 3 MB with efficacy shown both in cell lines in vitro and in mouse models in vivo [14, 16, 36, 48, 49]. It might be of great interest to explore similar treatment regimens in SMARCA4-deficient MB, especially since the response could differ significantly. For example, Romero et al. have shown that SMARCA4-deficient lung cancer cells do not respond to HDAC inhibition but in contrast are sensitive to inhibition of the demethylases KDM6A/B, even if MYC is concurrently amplified [56]. This observation emphasizes the importance of considering alternative treatment options for SMARCA4-deficient MB.

## Conclusions

For the first time, we showed cooperative effects between MYC overexpression and a SMARCA4 loss in driving tumorigenesis in cerebellar precursors. Tumors displayed histological and molecular resemblance to Group 3 MB with a distinct selection for SMARCA4-deficient cells. Taken together, these findings provide evidence for a tumor-promoting role of a *SMARCA4* deficiency in Group 3 MB. Consequently, our observations pave the way for further investigations on SMARCA4-deficient MB mouse models with the potential to identify therapeutic targets specific to these frequently occurring alterations.

### Electronic supplementary material

Below is the link to the electronic supplementary material.

Additional File 1

Additional File 2

## Data Availability

The datasets generated and analyzed within this study are available in the GEO repository, https://www.ncbi.nlm.nih.gov/geo/, accession numbers GSE235625 (RNA sequencing data) and GSE235924 (DNA methylation data). Other data and material described in this study are available from the corresponding author upon request.

## List of Abbreviations

ATRT: Atypical/teratoid rhabdoid tumor
BAF: BRG1/BRM-associated factor
BrdU: Bromodeoxyuridine
BRG1: BRAHMA related gene 1
CC3: Cleaved-Caspase 3
FDR: False Discovery Rate
FFPE: Formalin-fixed paraffin-embedded
GAPDH: Glyceraldehyde 3-phosphate dehydrogenase
GCP(s): Granule cell precursor(s)
IF: Immunofluorescence
IHC: Immunohistochemistry
LCA: Large cell/anaplastic
MB: Medulloblastoma
NSCLC: Non-small cell lung cancer
OLIG2: Oligodendrocyte transcription factor 2
rRNA: Ribosomal RNA
SCCOHT: Small cell carcinoma of the ovary, hypercalcemic type
SHH: Sonic Hedgehog
SMARCA4: SWI/SNF related, matrix associated, actin dependent regulator of chromatin, subfamily A, member 4
SOX2: Sex determining region Y
Tam: Tamoxifen
UMAP: Uniform Manifold Approximation and Projection for Dimension Reduction
WNT: Wingless/Int-1
