# Supplementary material for: MYC overexpression and SMARCA4 loss cooperate to drive medulloblastoma formation in mice
Source: Acta Neuropathol Commun. 2023 Nov 2;11:174. doi: 10.1186/s40478-023-01654-2 (PMC10621315; doi:10.1186/s40478-023-01654-2)
Supplement: Supplementary file 1 — Additional File 1 [file 40478_2023_1654_MOESM1_ESM.pdf]

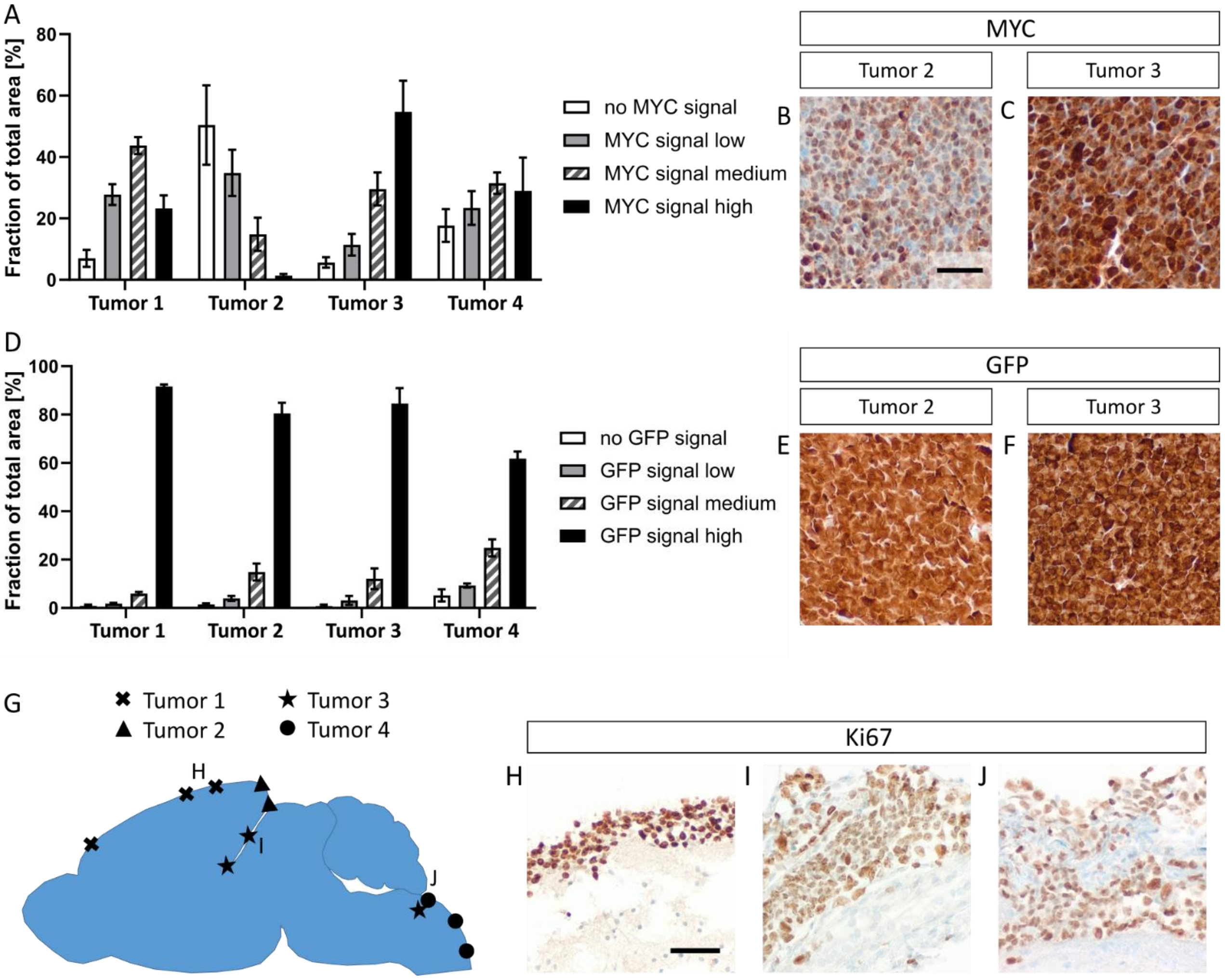

Figure S1. MYC/SMARCA4 tumors exhibit intratumoral heterogeneity of MYC expression and leptomeningeal spread within the brain. (A) Fraction of total area showing no, low, medium, or high MYC signal in DAB staining within five pictures of each MYC/SMARCA4 tumor taken from different regions within the tumor. Tumor 5 was excluded from the analysis due to its small size. (B,C) Exemplary pictures of MYC DAB stainings in tumors 2 and 3 displaying varying degrees of staining intensities including cells without any MYC signal. (D) Fraction of total area showing no, low, medium, or high GFP signal in DAB staining within five pictures of each MYC/SMARCA4 tumor. (E,F) Exemplary pictures of GFP DAB stainings in tumors 2 and 3 showing an uniformly high signal. (G) Illustration of a sagittal brain section indicating regions where leptomeningeal spread was detected as defined by the presence of Ki67-positive SMARCA4-deficient cells. Tumor 5 did not display any leptomeningeal spread. (H-J) Exemplary images of leptomeningeal spread in the regions indicated in G, staining positive for Ki67. Scale bars correspond to 50 µm in B (also applicable to C,E,F) and to 50 µm in H (also applicable to I+J).

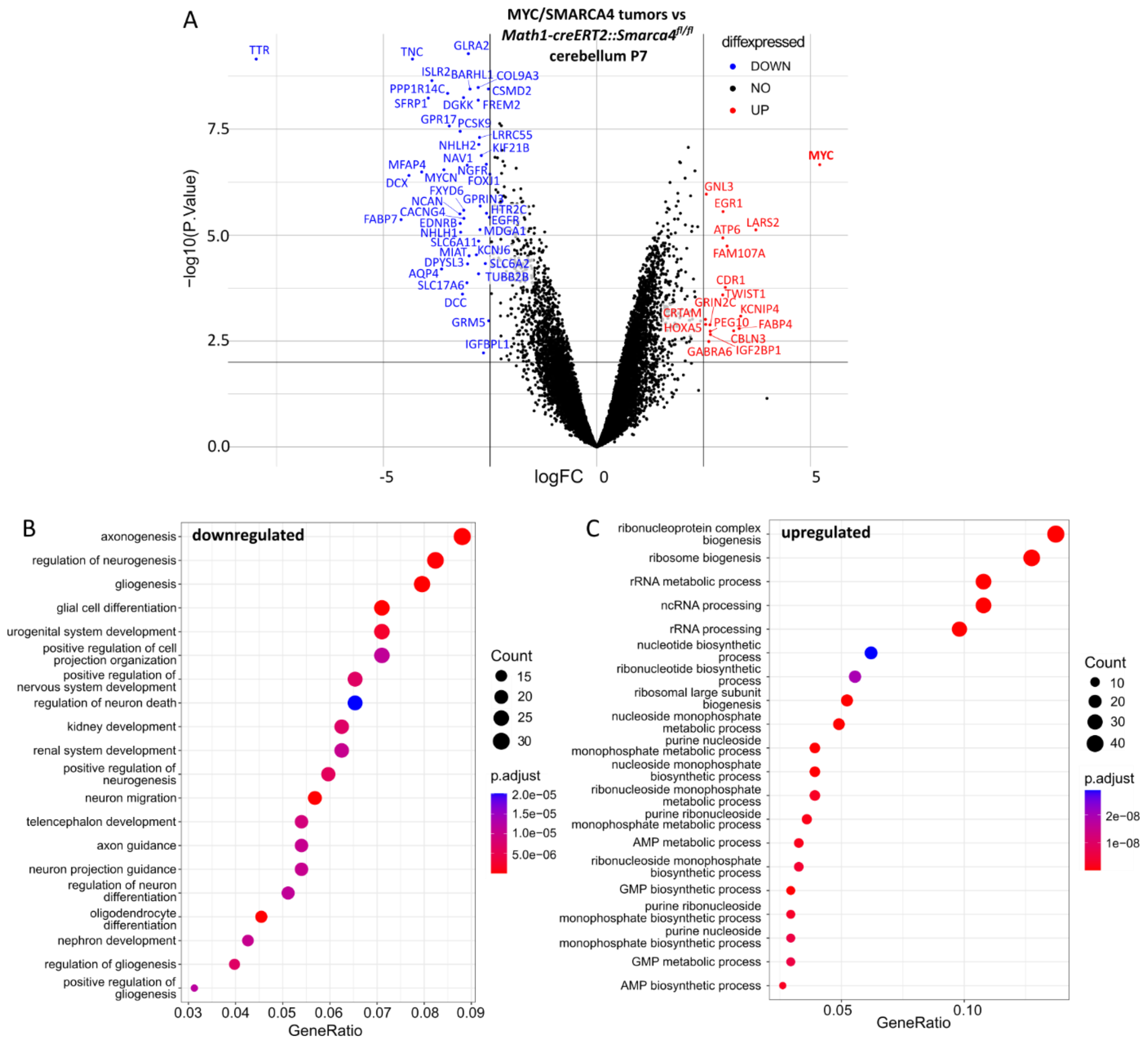

Figure S2. Differential gene expression of MYC/SMARCA4 tumors compared to *Math1-creER^{T2}::Smarca4$^{fl/fl}$* cerebella at P7. (A) Volcano plot depicting differential gene expression as assessed by RNA sequencing analysis between our MYC/SMARCA4 tumor model (n=4) and P7 cerebella of *Math1-creER^{T2}::Smarca4$^{fl/fl}$* mice after tamoxifen injection at P3 (n=3), recapitulating the cell of origin for our tumors. Only genes orthologous in mouse and humans were visualized, and differential expression with logFC ≥ 2.5 and p ≤ 0.01 was considered significant (blue/red coloring) after Benjamini-Hochberg correction. (B,C) Gene set enrichment analysis was performed based on significantly differentially expressed genes considering all mouse genes with logFC ≥ 1.5 and p ≤ 0.01.
